# Rapid and Efficient Detection of the SARS-CoV-2 Spike Protein Using an Electrochemical Aptamer-Based Sensor

**DOI:** 10.1021/acssensors.1c01222

**Published:** 2021-08-10

**Authors:** Andrea Idili, Claudio Parolo, Ruslán Alvarez-Diduk, Arben Merkoçi

**Affiliations:** † Institut Català de Nanociència i Nanotecnologia (ICN2), Campus UAB, Bellaterra 08193 Barcelona, Spain; ‡ Barcelona Institute for Global Health, 08036 Barcelona, Spain; § CSIC and the Barcelona Institute of Science and Technology (BIST), 08036 Barcelona, Spain; ∥ Institucio′ Catalana de Recerca i Estudis Avançats (ICREA), 08010 Barcelona, Spain

**Keywords:** aptasensors, electrochemical sensors, infectious diseases, EAB sensors, COVID-19

## Abstract

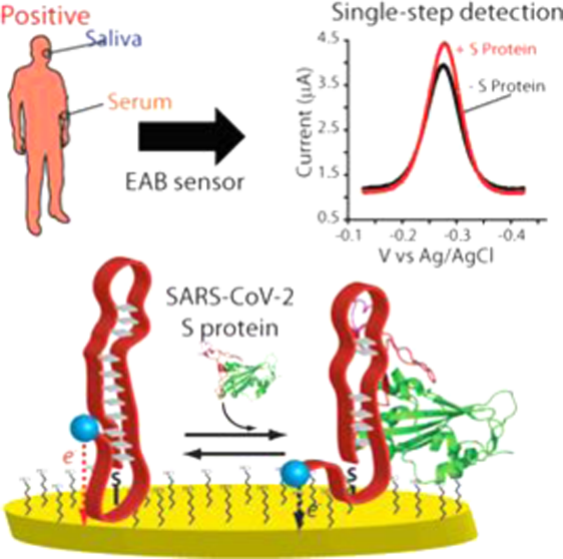

The availability of sensors able to rapidly detect SARS-CoV-2 directly in biological fluids in a single step would allow performing massive diagnostic testing to track in real time and contain the spread of COVID-19. Motivated by this, here, we developed an electrochemical aptamer-based (EAB) sensor able to achieve the rapid, reagentless, and quantitative measurement of the SARS-CoV-2 spike (S) protein. First, we demonstrated the ability of the selected aptamer to undergo a binding-induced conformational change in the presence of its target using fluorescence spectroscopy. Then, we engineered the aptamer to work as a bioreceptor in the EAB platform and we demonstrated its sensitivity and specificity. Finally, to demonstrate the clinical potential of the sensor, we tested it directly in biological fluids (serum and artificial saliva), achieving the rapid (minutes) and single-step detection of the S protein in its clinical range.

The current COVID-19 pandemic has made it clear how a highly infectious airborne pathogen (such as SARS-CoV-2) has the ability to spread globally in a matter of weeks.^1,2^ As influenza viruses, SARS-CoV-2 travels from patient to patient within respiratory droplets (e.g., small aqueous particles of saliva or mucus produced by exhalation), making it extremely challenging to contain due to its close relationship with social distance (Figure 1A).^3,4^ Once the virus gets inside the organism, its membrane proteins (i.e., spike (S) protein) interact with the transmembrane ACE2 receptor infecting the host’s cells. The binding between the two proteins triggers the cellular fusion of the virus and subsequent release of its genetic material into the cytosol.^2,5,6^ This, in turn, allows the virus to replicate inducing tissue inflammation and leading to shortness of breath, chest pain, loss of speech, and eventually death (Figure 1A).^2,7^ Besides the dramatic effects that this virus can have on single individuals, we have also seen how a pandemic can affect the entire world population by limiting movements and having a huge impact on the global economy.^8,9^ So how can we deal with such a catastrophe? Unfortunately, like any other disease, finding the vaccine and cure need their time (in the case of vaccine following the accelerated protocol, it took at least one year); instead, specific diagnostic devices are available in weeks, providing monitoring tools to limit the spread of the disease (e.g., by introducing lockdowns and evaluate the efficiency of prophylactic actions).^9−11^

**Figure 1:**
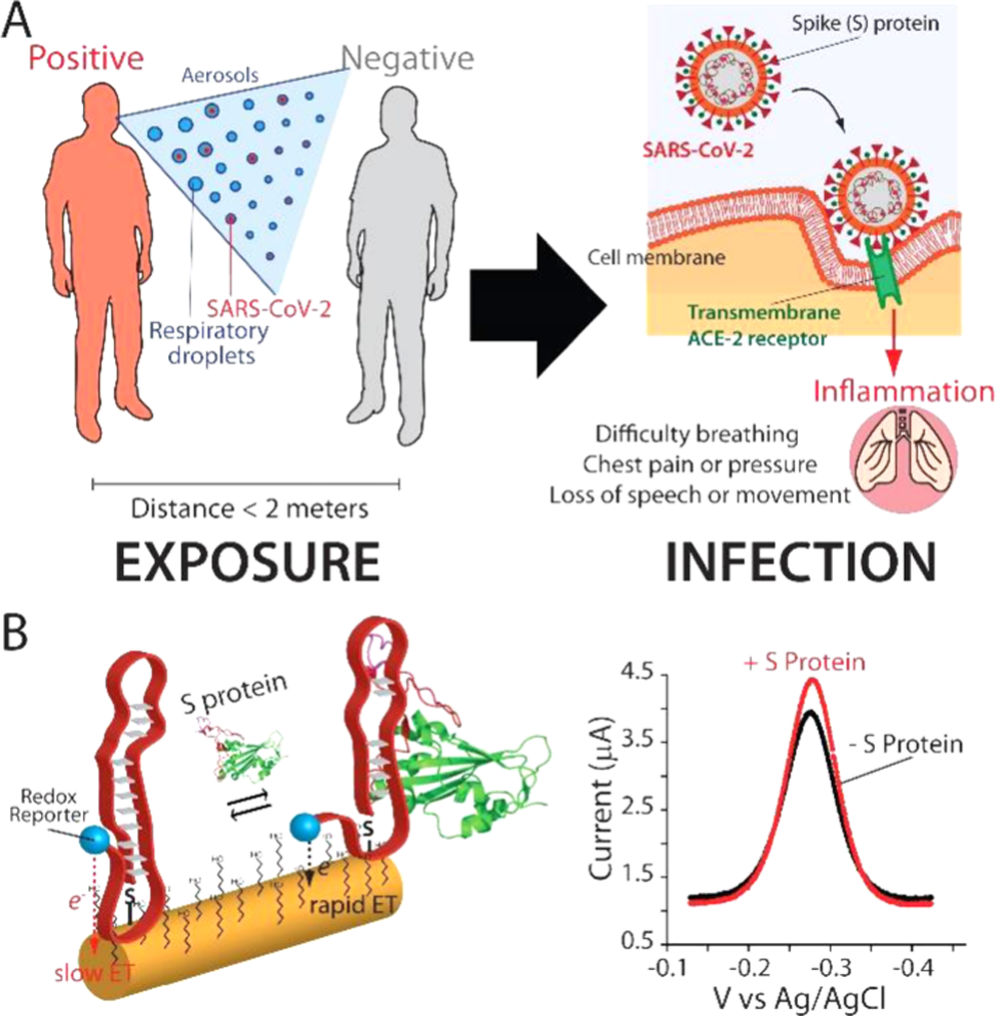
Ongoing COVID-19 pandemic is a direct effect of the appearance and the rapid global spreading of severe acute respiratory syndrome coronavirus 2 (SARS-CoV-2).^19^ (A, left) The main human-to-human transmission route is driven by respiratory droplets produced by the infected person (here, represented as the red person).^2^ The inhalation of these droplets allows the transmission of the virus to another individual (blue healthy person). More specifically, (A, right) SARS-CoV-2 can travel deeply in the respiratory tract until reaching the lungs. There, because the viral particles cannot be recognized by the immune system,^20^ they start to interact with the cells through their membrane spike (S) proteins, which can be recognized by the angiotensin-converting enzyme 2 (ACE2) receptor. This binding first triggers the virus entry into the patient’s cell through the cleavage of the S1/S2 site by surface transmembrane protease serine 2,^2^ and the next activation of endolysosomal cathepsin L to induce virus–cell membrane fusion. After this event, the RNA genome is released into the cytosol of the patient’s cell, where it is translated into the proteins, which can activate the replication of the virus inside the patient’s cells. (B) Electrochemical aptamer-based (EAB) sensors exploit the binding-induced conformational change of a covalently attached, redox reporter-modified aptamer to generate an easily measurable electrochemical signal. Specifically, the changes in conformation alter the rate with which the redox reporter (here, we used Atto MB2, a derivative of methylene blue) exchange electrons with the interrogating electrode. Because this sensing mechanism is based on a binding-induced conformational change of the receptor, the platform results in reagentless, rapid, and selective measurements directly in undiluted biological fluids.^21^

Current molecular approaches to infectious disease diagnosis are not rapid and decentralized enough to keep up with the spread rate of a highly infectious pathogen in a globalized world.^12,13^ For example, the current gold standard technique used to diagnose COVID-19 is the polymerase chain reaction (PCR), which provides high sensitivity and specificity through the direct quantification of the viral RNA. This is a crucial clinical parameter to estimate the stage of the infection and to discover asymptomatic patients (i.e., people infected with COVID-19 that cannot be easily identified due to the absence of symptoms).^14^ Despite these clinical advantages, the PCR requires trained personnel, expensive equipment, delicate reagents, and a relatively long procedure, which hamper its use for frequent testing (multiple times per week)^15^ and in low-resource settings.^9,10,16^ These disadvantages make PCR too slow for the immediate identification of infected asymptomatic individuals, leading to delays in the application of containment measures allowing the virus to spread further.^15^ As an alternative to the PCR, lateral flow immunoassays (LFIAs) provide a more rapid response at the point of care,^17^ but their lower sensitivity and specificity relegate them primarily for end-point serologic applications (i.e., the detection of anti-SARS-CoV-2 antibodies weeks after getting infected).^18^ Even the most recent LFIAs for antigenic testing have considerably lower analytical performance (compared to the PCR), leading to delayed and qualitative diagnosis, which in turn affects the way patients are managed.^18^ Therefore, the development of biosensing platforms able to detect the clinically relevant concentrations of SARS-CoV-2 in a single step directly in untreated biological fluids could represent a new clinical tool to achieve rapidly and efficiently contact tracking of the COVID-19 outbreak.^15^

Recently, we and other researchers have been working on the development of a new type of sensing technology that can provide a precise quantitative response at the point of care: electrochemical aptamer-based (EAB) sensors.^22−27^ This technology relies on the signal produced by a binding-induced conformational change of a redox reporter-modified aptamer on a gold electrode surface (Figure 1B). A variation in the target concentration induces a change in the aptamer conformation, which in turn changes the position of the redox reporter (here, we used Atto MB2, which is a methylene blue derivative) relative to the electrode surface, generating a quantitative electrochemical signal.^28−32^ The precise analytical response of the EAB sensor is coupled with their quick response time (from few seconds to 5 min) and extremely simple operation (single step), making them ideal diagnostic devices for COVID-19 monitoring through high-frequency testing. Motivated by this, here, we describe the development and characterization of a new EAB sensor against the SARS-CoV-2 S protein and its ability to recognize the target in undiluted samples (serum and artificial saliva) in its clinical range.

## Results and Discussion

As the recognition element for our sensor, we selected two recently developed DNA aptamers, termed 1C and 4C, able to recognize the receptor-binding domain (RBD) of the SARS-CoV-2 spike (S) protein.^33^ We selected these aptamers because they display three key features for the development of a responsive EAB sensor. First, the variants have been selected in a working buffer that mimics the physiological conditions; therefore, they can support the measurement of the S protein in biological fluids.^21^ Second, the estimated dissociation constants (*K*_D_) of the aptamers (5.8 ± 0.8 nM for 1C and 19.9 ± 2.6 nM for 4C) are comparable to those of commercially available antibodies developed to bind the S protein.^34^ Third, the binding interactions between the target and the aptamers have been previously characterized through the molecular dynamics (MD) technique.^33^ More specifically, the variants can interact with the S protein through the formation of a network of hydrogen bonds creating two consecutive binding interfaces in the case of the 1C variant, and only one for the 4C variant.^33^ These data suggest that the magnitude of the binding-induced conformational change in the 1C variant is stronger than that in the 4C variant. Although this promising structural prediction confirms the binding, it is not clear if these interactions can induce a conformational change in the aptamers. Indeed, this structure-switching property is crucial to generate a detectable output signal on the EAB sensing platform (Figure 1B).^28,35^

**Figure 2:**
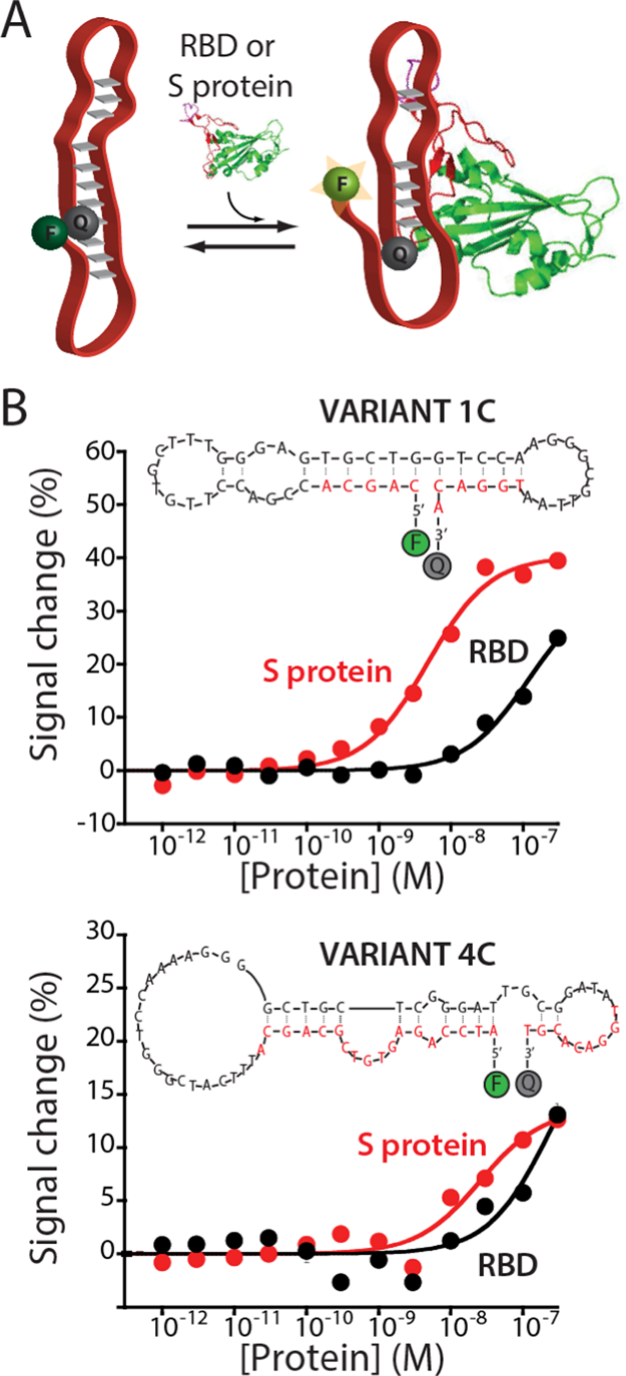
(A) We characterized the binding activities of aptamers against the SARS-CoV-2 RBD (black curves) and the S protein (red curves) in solution using fluorescence spectroscopy. To do this, we labeled the 1C and 4C variants with a fluorophore–quencher couple at the two ends. In the absence of the target, the close proximity between the fluorophore and the quencher decreases the overall fluorescent signal. Instead, the presence of the target induces a binding-induced conformational change in the aptamer structure, separating the fluorophore from the quencher, thus increasing the fluorescent signal. (B) We find that the 1C aptamer exhibits a lower dissociation constant (4.8 ± 0.8 nM) and a higher signal gain (+40.3 ± 1.5%) for the S protein (red curves) than the C4 variant (*K*_D_ = 25.4 ± 10.5 nM; 13.6 ± 1.5%). As expected, the optically labeled variants can also recognize also the RBD target (black curves) but they are displaying lower affinities and signal gain due to the smaller size of the target. The binding curves were obtained by adding increasing concentrations of protein targets to a 5 nM concentration of 1C (B, top) or 4C variant (B, bottom) in 0.1× PBS buffer (NaCl 13.7 mM, KCl 0.27 mM, Na_2_HPO_4_ 1 mM, and KH_2_PO_4_ 0.18 mM), 2 mM MgCl_2_, pH 7.4 at 25 °C.

We used fluorescence spectroscopy to study if the interaction with the target induces a conformational change in the selected aptamers. Because the binding event should bring their 5′-ends far away from the 3′-ends (with respect to their folded stem-loop state), we modified these termini with a pair of a fluorophore (6-FAM) and a quencher (BHQ-1) to track this structural variation (Figure 2A).^36,37^ As targets, we have used the RBD and the S protein for two reasons: (1) both proteins share the same target protein domain recognized by the aptamers and (2) the detection of the larger S protein could demonstrate the ability of the aptamers to support the recognition of the virus. To characterize them, we monitored the fluorescence signal of the aptamers in the presence of increasing concentrations of the targets (Figure S1). We found that the presence of the S protein induces an increase in the fluorescence signal, supporting the hypothesis that formation of the target–aptamer complex brings the fluorophore far away from the quencher. On the contrary, for the smaller RBD protein, we observe only a small suppression of the signal (Figure S1). We believe that this difference depends on the different sizes of the proteins and their ability to interact with their targets (e.g., ACE2 receptor) mainly through electrostatic and van der Waals interactions.^38,39^ Specifically, the S protein (78 kDa vs 23 kDa of the RBD) can interact with the negatively charged aptamer through a higher binding interface, which could generate a different microenvironment, for example, in the presence of extra charged amino acids located in the proximity of the bound oligonucleotide. This could induce a stronger conformational change in the aptamer structure. Fitting the collected data using a Langmuir isotherm equation (see the Materials and Methods section), we can estimate with precision the *K*_D_ associated with the S protein. Specifically, we find *K*_D_ values of 26 ± 8 nM for the 1C variant and 11 ± 5 nM for the 4C variant and both aptamers display a comparable signal change. However, we observe that the estimated affinities have slightly higher values than what was previously reported.^33^ We suspect that this could be due to the presence of the fluorophore–quencher pair in their sequences. Specifically, previous studies have demonstrated how the formation of the fluorophore–quencher complex could stabilize the native structure of DNA-based structures inducing a more folded conformation.^40−42^

To better understand the different binding behaviors observed with respect to the previous study, we have reduced the stability of the native conformation of the aptamers used for the fluorescent studies. Specifically, by reducing the electrostatic interactions between mono- and divalent cations (present in the buffer solution) with the negatively charged phosphate backbone, we balanced the stabilization produced by the fluorophore/quencher pair.^40,41^ To achieve this, we have adopted a new working buffer with a lower salt content but with the same amount of magnesium ions (2 mM, see the Materials and Methods section for the details). We decided to adopt this strategy to avoid modification of the aptamer sequences, which could negatively affect the variants’ binding activities. Then, to demonstrate the reduced stability of the aptamers we used thermal melting curve experiments. This apporach allows us to associate the aptamer stability with respect to its melting temperature (*T*_M_) value.^43^ Basically, we observed at which temperature and buffer solution 50% of the aptamer population unfolds due to the thermal denaturation process. This induces the fluorophore and the quencher to move apart leading to an increase in the fluorescence signal. Therefore, a lower or higher *T*_M_ value can be associated with the lower or higher thermodynamic stability of the aptamer variants. As expected, we found both variants display lower melting temperatures when they are tested in the new buffer supporting the validity of our approach (Figure S2).

Next, we tested again the bioreceptors in the presence of target proteins (Figures 2B and S3). We observe for both variants, a higher positive fluorescence signal change and lower *K*_D_s. Specifically, the 1C variant displays maximum signal gain values of +40.3 ± 1.5 and +33.9 ± 3.8% and *K*_D_ values of 4.8 ± 0.8 and 116 ± 34 nM for the S protein and the RBD (Figures 2B, top and S3), respectively, while the 4C variant displays signal gain values of +13.6 ± 1.5 and +13.1 ± 5.0% and *K*_D_ values of 25.4 ± 10.5 and 24 ± 23 nM for the S protein and the RBD (Figures 2B, bottom and S3), respectively. The observed signal change further confirms the ability of the aptamers to undergo a binding-induced conformational change. Finally, since the 1C aptamer displays the higher signal gain and the lower *K*_D_ for the S protein, we selected this variant for its next adaptation as a bioreceptor for the EAB sensing platform. To this purpose, we replaced the fluorophore and the quencher with a thiol group at 5′-ends and Atto MB2 (a methylene blue derivative) at 3′-ends as a redox tag (Figure S4).

The newly fabricated EAB sensor responds to the presence of the target protein when interrogated using square wave voltammetry (SWV, Figure S5). To demonstrate the ability of the aptamer to undergo a binding-induced conformational change upon its attachment on the gold electrode, we characterized the relationship between its signal change and the frequency of the interrogating SWV potential pulse.^44^ Previous studies^25,26,45−47^ clearly demonstrate that the SWV frequency used during the sampling of the electrochemical signal is crucial for the optimization of EAB sensors. Specifically, this parameter affects the sign of the signal change (i.e., generating either signal-off or signal-on behavior) and its magnitude (overall signal gain). This is mainly due to the binding-induced changes in aptamer flexibility, which alters its electron transfer kinetic because the redox reporter could be pushed farther from or closer to the electrode surface upon binding with the target. Therefore, to explore this, we tested our sensor in the presence of a saturating concentration of the RBD (100 nM), and we collected its electrochemical response over square wave frequencies ranging from 5 to 1000 Hz (Figure S5). We find that the sensor’s signal displays a strong dependency on the SWV frequency. Specifically, we observed at a high frequency a signal-on response and at a low frequency a signal-off response. This dual behavior further demonstrates the presence of a binding-induced conformational change since it indicates the presence of two major aptamer conformation populations.

**Figure 3:**
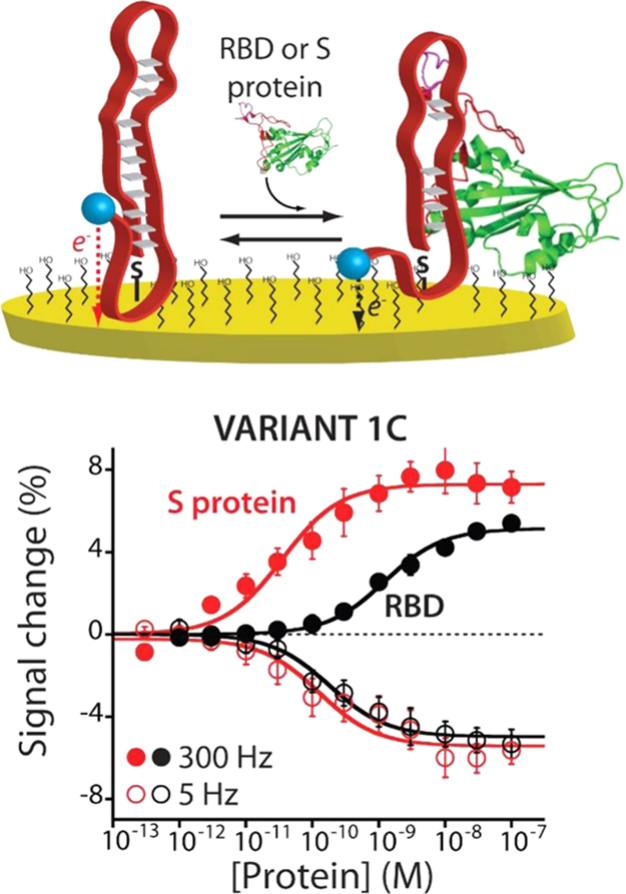
Newly fabricated EAB sensor responds to the increasing concentrations of the SARS-CoV-2 RBD (black curves) and the S protein (red curves) producing the expected Langmuir binding curves. To support the electrochemical readout, we modified the aptamer with an Atto MB2 redox reporter and attached it via a hexanethiol anchor to a gold wire electrode coated with a self-assembled monolayer of 6-mercapto-1-hexanol. As previously observed for sensors belonging to this class, our EAB sensor displays a signal-on response at higher square wave frequencies (300 Hz) and a signal-off response at lower frequencies (5 Hz). At the same time, the dynamic range (defined here as the range from 10 to 90% of the maximum signal change) of the sensor allows covering the clinically relevant range of SARS-CoV-2 in infected individuals,^48^ demonstrating its clinical value. The binding curves were obtained in PBS buffer, 2 mM MgCl_2_, pH 7.4 at 25 °C, using square wave voltammetry (SWV). The error bars reflect standard deviations derived using at least three independently fabricated sensors.

Our EAB sensors can recognize the S protein in its clinically relevant range, providing a new analytical tool for the detection of SARS-CoV-2. To characterize our sensors, we recorded their SWV signal in the presence of increasing concentrations of the RBD and the S protein (Figure 3). We observe for both targets the expected Langmuir isotherm binding curves at all square wave frequencies tested (5 and 300 Hz). Comparing the collected data, we note that the S protein produces a slightly larger response than the RBD. Specifically, the former target displays maximum signal gain values of −5.2 ± 0.4 and of +7.3 ± 0.5% at 5 and 300 Hz, respectively, while the latter displays signal gain values of −5.0 ± 0.2 and +5.1 ± 0.2% (Figure 2), respectively, for frequencies at 5 and 300 Hz. To understand the clinical value of the sensor, we estimated the associated dissociation constants through the analysis of the binding curves (see the Materials and Methods section). We found that the aptamer displays different affinities or *K*_D_s for the two targets. Specifically, the RBD results in *K*_D_ values of 181 ± 43 and 1231 ± 209 pM at 5 and 300 Hz, respectively, which is in agreement with the previous study (Figure 2).^34,49^ The larger S protein displays a better sensitivity and *K*_D_ values of 35.4 ± 11.7 and 126.4 ± 50.4 pM at 300 and 5 Hz, respectively, which cover the wide clinical relevant range.^34,50^ More specifically, although the sensor is not able to achieve the same sensitivity of molecular techniques based on enzymatic amplification such as PCR or LAMP (from 1 to 100 copies/mL),^51^ its dynamic range cover a concentration range (from ≈760 pg/mL to 76 ng/mL) that is comparable with point-of-care approaches developed for the detection of SARS-CoV-2 and used for high-frequency testing.^52,53^ Moreover, we find that the estimated *K*_D_ for the 1C variant on the surface is lower than what we have obtained using fluorescence spectroscopy (Figure S3). We believe that this result could arise from two additive effects. The first one is originated from the different labeling molecules used to modify the aptamer variant and track its conformational change. As highlighted before, the fluorophore–quencher couple can stabilize the native conformation of the aptamer^40−42^ with respect to the redox molecule that does not affect its stability.^54^ The second effect arises from the ability of the larger S protein to strongly interact with surfaces through electrostatic and van der Waals interactions (i.e., not selected during the SELEX process). These combined effects lead to an improvement of the overall affinity of the variant on the electrode surface.^55−57^

**Figure 4:**
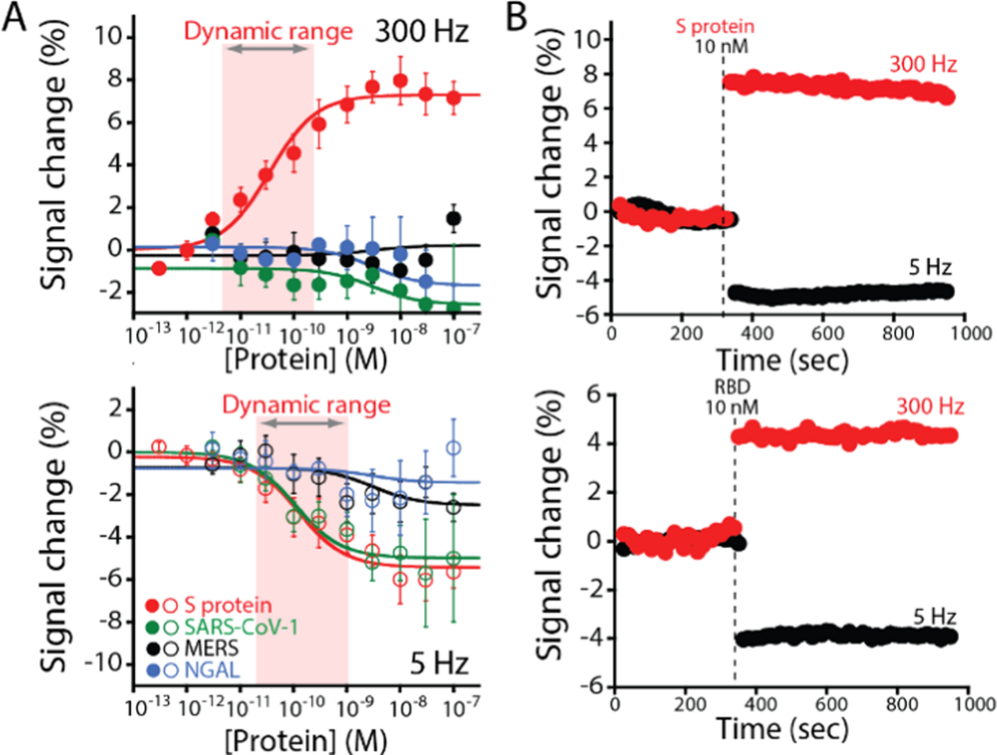
Sensor is specific and rapid providing a new clinical tool for the detection of the SARS-CoV-2 S protein. (A) To demonstrate this, we challenged the EAB sensor with the viral protein SARS-CoV-1 RBD (green curves) and MERS RBD (black curves) and the protein biomarker NGAL (blue curves). At 300 Hz (A, top), we do not observe a detectable signal change from any interference proteins over the concentration range we tested. On the contrary, at 5 Hz, the SARS-COV RBD is the only protein able to induce a response in the sensor, which is similar to the SARS-CoV-2 S protein (red curves). We believe that this result is due to the similarity of the proteins’ native structure and the lack of a counter-selection round during the SELEX process. The error bars reflect standard deviations derived using at least three independently fabricated sensors. (B) The sensor is rapid and can respond to the protein target in less than 20 s upon the addition of a clinically relevant concentration of the SARS-CoV-2 S protein (top) and the RBD (bottom). The electrochemical signal was collected at 300 Hz (red) and 5 Hz (black) square wave frequencies. The binding curves and kinetic experiments were obtained in PBS buffer, 2 mM MgCl_2_, pH 7.4 at 25 °C, using square wave voltammetry (SWV).

Our EAB sensor displays enough specificity to recognize the S protein from different coronaviruses, indicating its potential use for diagnostic applications. We tested the selectivity of the sensor challenging it with two RBD proteins selected from previous coronaviruses (SARS-CoV and MERS-CoV) and the non-viral protein NGAL (a protein found in various bodily fluids^27^ including saliva, blood, and urine). We observe that the sensor at 300 Hz does not produce a detectable signal change for any of the three proteins over the range of concentrations tested (from 10 pM to 100 nM) (Figure 4A, top). On the contrary, at lower SWV frequencies (5 Hz), only the SARS-CoV-1 RBD protein induces a signal change of the sensor, which is comparable to what is observed for SARS-CoV-2 (Figure 4A, bottom). We believe that this result depends on the selected aptamer and the structural properties of the target. Specifically, the aptamer was selected without performing a counter-selection against the SARS-CoV RBD protein during the SELEX process,^33^ and at the same time the RBD target from SARS-CoV shares almost 70% of the overall structure with the SARS-CoV-2 RBD.^58−61^ Taking together these two conditions, we believe that the SARS-CoV-1 RBD protein is able to bind the aptamer receptor without inducing a conformational change; therefore, it can only decrease the collisions between the variant and the electrode surface (through steric hindrance), leading in a signal-off behavior. Despite this, the different signal responses at low and high SWV frequencies displayed by the sensor can be used to discriminate the different RBDs from SARS-CoV-2 and SARS-CoV-1 through a single measurement at 300 Hz or through a double detection at both frequencies. Therefore, our sensing platform displays all its potential to discriminate different S proteins variants and this could be improved only by the introduction of counter-selection steps during the selection of the aptamer to further improve its specificity.

The sensor response is so fast that can recognize the SARS-CoV-2 RBD target in seconds, demonstrating its ability to support point-of-care (PoC) sensing strategies. To evaluate the resolution time of the sensor, we exploited the ability of EAB sensors to perform high-frequency measurements. We collected the SWV signals at both frequencies (5 and 300 Hz), and after achieving a stable baseline (approximately after 5 min), the sensor was challenged with 10 nM S protein (Figure 4B, top). The sensor responds within 15 s from the addition of the target, which corresponds to the required time to collect the two voltammograms. To further demonstrate the rapidity of the binding kinetics, we challenged the sensor also with the RBD protein. Again, the sensor promptly responded to the injection of the RBD protein within 15 s, which demonstrates that the sensor’s response is not affected by the target’s size (Figure 4B, bottom).

**Figure 5:**
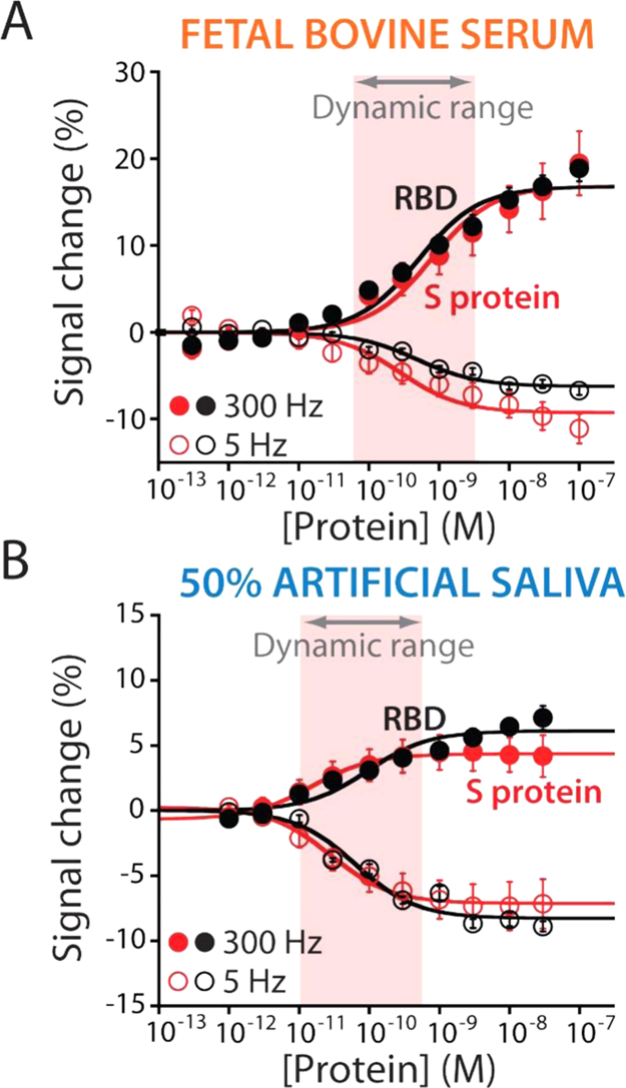
Our EAB sensors can fully support the detection of the SARS-CoV-2 S protein and the RBD directly in biological fluids, demonstrating all their potential to perform frequent testing. When the EAB sensors are challenged in (A) fetal bovine serum (FBS) and (B) 50% artificial saliva, they can detect both viral protein targets, displaying measurements with picomolar precision. The error bars reflect standard deviations derived using at least three independently fabricated sensors.

Because the signal transduction mechanism is based on the binding-induced conformational change of the recognition element, EAB sensors work well when used directly in untreated biological fluids in vitro and in vivo.^27,62,63^ Harnessing this unique feature, we tested our sensor directly in artificial saliva and fetal bovine serum (FBS) (Figure 5). We selected these biological fluids because the former is routinely used for COVID-19 diagnostics (e.g., PCR, antigenic tests, and cellular culture) due to the presence of the high concentration of viral particles,^64,65^ whereas the latter allows studying the EAB sensor response in extremely complex media. When the sensor is employed in undiluted FBS (Figure 5A), we observe maximum signal gain values of −9.2 ± 0.9 and +16.0 ± 1.3% at 5 and 300 Hz, respectively, and *K*_D_ values of 300 ± 100 and 800 ± 200 pM at 5 and 300 Hz, respectively. Although the estimated affinities are lower than those obtained using the buffer solution, the sensor’s dynamic range still covers the clinically relevant range.^34,48,50^ We believe that this difference arises from the fouling effect, which reduces the formation of electrostatic and van der Waals interactions between the target and the sensing surface.^57^ Then, the sensor was tested in 50% artificial saliva (Figure 5B). Specifically, at 5 Hz, the sensor still responds to the target with a maximum signal gain of −7.3 ± 0.2% and an estimated *K*_D_ of 28.2 ± 5.0 pM. At 300 Hz, we estimated a maximum signal gain of +5.0 ± 0.2% and an estimated *K*_D_ of 14.9 ± 2.0 pM. Motivated by the results, we further characterize our EAB sensor in undiluted artificial saliva (Figure S6) to understand if it could be translated to work in real saliva samples. Using this condition, the sensor is still able to detect the presence of the protein target in its clinical range; however, it displays a lower signal gain. We believe that the lower response is ascribed to the effect of the matrix on the aptamer’s conformational change. This suggests the need for an optimization study for its next employment for clinical or precommercial applications. Although biological fluids such as saliva could have an impact on the performance of the EAB sensor, the overall data demonstrate that our developed sensor could be used as a diagnostic tool for the rapid detection of the SARS-CoV-2 S protein.

## Conclusion

In this paper, we described the development of a new EAB sensor able to detect the SARS-CoV-2 S protein rapidly and efficiently in biological fluids. First, we characterized the binding activity of the selected aptamers for the S protein using fluorescence spectroscopy. Through the use of optically labeled variants, we demonstrate that the aptamers can support a binding-induced conformational change mechanism, making them ideal candidates to support EAB sensing platform. The resulting EAB sensor showed comparable bioanalytical performance in comparison to other methods using antibodies^34,50,66^ and aptamers^67,68^ for the detection of SARS-CoV-2 antigens. Specifically, using the 1C variant, we detected picomolar levels of the S protein in buffer, serum, and 50% artificial saliva. The specificity of the results are also very promising, indicating the ability of the EAB sensor to discriminate between similar targets (RBD portions of other coronaviruses) and other proteins, besides being able to detect the target in undiluted serum and artificial saliva. Finally, the quick binding kinetics of the aptamer and its single-step operation allow the detection of the target in 15 s, making our EAB sensor ideal to support high-frequency testing at the point of care. Undoubtedly, other technologies that have been used for decades (e.g., LFA and ELISA)^17,69^ are still far ahead in terms of their application and commercial availability; however, with this work, we want to show the potential of EAB sensing as a valid alternative to the current PoC tests. Additionally, their ability to support calibration-free and dual-reporter approaches^70,71^ (i.e., allowing the direct quantification of the target) and their versatility to be coupled with mobile phones or portable electrochemical setups could represent an additional tool to achieve real-time epidemiology.^26,34^

The secondary goal of this study is to help the community during the design and development of aptamer-based sensors, specifically (but not limited to) those relying on the binding-induced conformational change. For example, looking in particular at the aptamer characterization, our results clearly reinforce the need to introduce in the SELEX process both counter-selection steps and the use of buffers with different ionic strengths. The former is crucial to select aptamers that specifically bind through a conformational change the target protein even in the presence of similar contaminants. For example, the possibility to discriminate between the RBD portions of SARS-CoV-1 and SARS-CoV-2 would allow the precise diagnosis of similar yet different diseases, requiring different treatments and containing actions.^72^ The latter is important to guarantee the binding to the target in natural conditions, as it could be in a highly ionic strength environment such as saliva. Nonetheless, even with a suboptimal selection, the aptamers we tested showed excellent binding performance as bioreceptors in an EAB sensor. On the one hand, the use of different sampling frequencies allowed for the discrimination of the RBD from different coronaviruses. On the other hand, a simple dilution step (correcting the ionic strength of the matrix) allowed the detection of the target protein in artificial saliva.

## Supporting Information Available

Materials and methods; binding and melting curves for optically labeled aptamer variants; frequency map; and calibration curves performed in 100% saliva of the EAB sensor (PDF)
