# Supplementary material for: Rapid and Efficient Detection of the SARS-CoV-2 Spike Protein Using an Electrochemical Aptamer-Based Sensor
Source: ACS Sens. 2021 Aug 10;6(8):3093–101. doi: 10.1021/acssensors.1c01222 (PMC8370117; doi:10.1021/acssensors.1c01222)
Supplement: Supplementary file 1 — se1c01222_si_001.pdf [file se1c01222_si_001.pdf]

Supporting Information

Rapid and efficient detection of SARS-CoV-2 Spike protein using an electrochemical aptamer-based sensor

Andrea Idili,[a] Claudio Parolo,[a],[b] Ruslán Alvarez-Diduk,[a] and Arben Merkoçi*[a], [c], [d]

[a] Institut Català de Nanociència i Nanotecnologia (ICN2), Campus UAB, Bellaterra, 08193 Barcelona, Spain
[b] Barcelona Institute for Global Health,08036 Barcelona, Spain
[c] CSIC and the Barcelona Institute of Science and Technology (BIST), 08036 Barcelona, Spain
[d] Institucio´ Catalana de Recerca i Estudis Avanc¸ats (ICREA), 08010 Barcelona, Spain
E-mail: arben.merkoci@icn2.cat

Table of Contents

Materials and Methods S3

Figure S1. Binding curves of the optically labeled aptamers performed in working buffer S6

Figure S2. Melting curves of the aptamer variants in the absence of the target using different buffers S7

Figure S3. Binding curves of the optically labeled aptamers performed in a new buffer with a lower salt content S8

Figure S4. Sequence of the selected aptamer and its modifications to support electrochemical readout S9

Figure S5. Frequency map of the newly fabricated EAB sensor S10

Figure S6. Binding curve performed in 100% artificial saliva S11

References S12

Materials and Methods

Chemical reagents and materials. Reagent-grade chemicals, including phosphate buffered saline tablets (PBS), sodium hydroxide (NaOH), hydrochloric acid (HCl), 6-mercapto-1-hexanol, sodium chloride (NaCl), ethanol (CH_3CH_2OH), magnesium chloride ($MgCl_2$), tris(2-carboxyethyl)-phosphine hydrochloride (TCEP), tris[hydroxymethyl]-aminomethane hydrochloride (Trizma), ethylenediaminetetraacetic acid (EDTA), and Fetal Bovine Serum were obtained from Sigma-Aldrich (St. Louis, MO, USA), and they were used as received. Sulfuric acid 96% (H_2SO_4) was obtained from Panreac Química S.L.U. (Barcelona, Spain). Protein targets including SARS-CoV-2 (2019-nCoV) Spike RBD Recombinant Protein (25.1 kDa), SARS-CoV-2 (2019- nCoV) Spike S1(D614G)-His Recombinant Protein (76.41 kDa), SARS-CoV Spike/RBD Protein (RBD, mFc Tag) (51.4 kDa), MERS-CoV Spike/RBD Protein fragment (RBD, aa 367-606, His Tag) (27.7 kDa), and Recombinant Human Lipocalin-2 (NGAL) (25 kDa) were obtained from Sino Biological Europe GmbH (Eschborn, Germany) and they were received lyophilized. To dissolve the proteins, we added in the tube 400 uL of sterilized water to reach a final concentration of 0.25 mg/mL and then aliquot and stored at -80 °C. Artificial Saliva for Medical and Dental Research (Part number 1700-0305) was purchased from LCTech GmbH (Obertaufkirchen, Germany) and it was used as received. The fritted Ag|AgCl electrodes (CHI111/112 reference electrode) and platinum wire (CHI115 -counter electrode) were purchased from CH Instruments (Austin, TX, USA).

Oligonucleotides. RP-HPLC purified oligonucleotides were purchased from Biomers GmbH (Ulm, Germany). The aptamer sequences used for the optical characterization (Figures 2, S1 and S2) were modified with the fluorophore 6-FAM at their 5' end and the quencher BHQ-1 at their 3' end. The aptamer 1C variant to support the electrochemical characterization (Figure 3, 4, 5, S5, and S6) was modified with a thiol-C_6-SS group at its 5' end, and an Atto MB2 at 3' end (Figure S3).[1] More specifically, this redox tag is a methylene blue azide molecule attached to the aptamer sequence with 5'-ACH linker. The oligonucleotides were dissolved in TE buffer (100 mM Tris buffer, 10 mM $MgCl_2$, pH 7.8) at a concentration of 100 μM and then aliquot and stored at -20 °C. The final concentration of the oligonucleotides was confirmed using a Shimadzu mod. UV-1900 Spectrophotometer (Duisbuirg, Germany) using a TrayCell (optical path 0.02 cm) and measuring the relative absorbance at 260 nm. The aptamer sequences we employed in this work have been previously reported:[2]

1C variant: 5'- CAGCA-CCGAC-CTTGT-GCTTT-GGGAG-TGCTG-GTCCA-AGGGC-GTTAA-TGGAC-A-3'

4C variant: 5'-ATCCA-GAGTG-ACGCA-GCATT-TCATC-GGGTC-CAAAA-GGGGC-TGCTC-GGGAT-TGCGG-ATATG-GACAC-GT-3'

Fluorescence experiments. Binding curves (Figures 2, S1, and S3) were obtained using a Varian Cary Eclipse Fluorometer with excitation at 490 (±5) nm and acquisition between 500(±5) nm and 600 (±5) nm at a temperature of 25˚C and using a total volume of 1000 μl in a quartz cuvette. The 1C and 4C optically-labeled variants were dissolved in the working buffer (NaCl 137 mM, KCl 2.7 mM, Na_2HPO_4 10 mM, KH_2PO_4 1.8 mM, $MgCl_2$ 2 mM at pH 7.4; Figure S1) and in its diluted version (NaCl 13.7 mM, KCl 0.27 mM, Na_2HPO_4 1 mM, KH_2PO_4 0.18 mM, $MgCl_2$ 2 mM, at pH 7.4; Figure 2 and S3) at a final concentration of 5 nM. We recorded the fluorescence signal of the aptamer variants in the absence of protein targets until a stable signal was obtained. We then added the RBD (stock solution 9.96 μM) or S1 protein (stock solution 3.33 μM) at various concentrations and, after 15 min under magnetic stirring, fluorescence spectra were recorded. The fluorescence intensity at 520 nm (corresponding to the maximum emission of 6-FAM) was used to generate binding curves (Figures 2, S1, and S3) and fitted using a single-site binding model:[3]

$$\boldsymbol{F_{Variant}^{[Target]}} = \boldsymbol{F_{Variant}^{0}} + \left(\frac{[\boldsymbol{Target}](\boldsymbol{F_{Variant}^{Target-MAX}} - \boldsymbol{F_{Variant}^{0}})}{[\boldsymbol{Target}] + \boldsymbol{K_D}}\right) \quad \text{Eq. 1}$$

Where [Target] is the concentration of the protein target, $\boldsymbol{F_{Variant}^{[Target]}}$ is the aptamer fluorescence signal in the presence of different concentrations of the target, $\boldsymbol{F_{Variant}^{0}}$ is the background fluorescence of aptamers in the absence of the target, $\boldsymbol{F_{Variant}^{Target-MAX}}$ is the aptamers' fluorescence signal in the presence of saturating concentrations of the protein target, and K_D is the dissociation constant of the binding process. Using the estimated values for $\boldsymbol{F_{Variant}^{0}}$ we converted the raw fluorescence to % signal change using the following equation:

$$F_{Variant}^{\%Signal} = \left(\frac{(F_{Variant}^{[Target]} - F_{Variant}^{0})}{F_{Variant}^{0}}\right) * 100 \quad \text{Eq. 2}$$

Thermal melting curves. Melting curve experiments for the 1C and 4C variant (Figure S3) were obtained using a Cary Eclipse Fluorimeter (Agilent Technologies), using a total volume of 1000 μL. The optically labeled variants were dissolved in the working buffer (NaCl 137 mM, KCl 2.7 mM, Na_2HPO_4 10 mM, KH_2PO_4 1.8 mM, $MgCl_2$ 2 mM at pH 7.4; Figure S1) and in its diluted version (NaCl 13.7 mM, KCl 0.27 mM, Na_2HPO_4 1 mM, KH_2PO_4 0.18 mM, $MgCl_2$ 2

mM, at pH 7.4; Figure 2 and S3) at a final concentration of 5 nM. Before their use, the solutions were heated to 95°C for 5 min and then allowed to cool into ice for 30 mins. Melting curves were performed using an excitation at 490 (± 5) nm and acquisition at 518 (± 5) nm (corresponding to the maximum emission of 6-FAM), and by heating from 20 °C to 95 °C at a rate of 1 °C·min^{-1}. All thermal melting curves were analyzed and fitted using Kaleidagraph (V. 4.5), a graphing and data analysis software. The collected raw fluorescence signals have been normalized through the use of the interpolation model.[4,5] Then, melting temperatures (T_M) have been obtained using the same model from the intersection of the calculated median and the experimental melting curve.[4,5]

Gold Wire Electrode Fabrication and Electrochemical Cleaning. The EAB sensors employed in our in-vitro characterization (Figure 3, 4, 5, S5, and S6) were fabricated using an established approach.[6] Briefly, segments of bare gold wire with a diameter of 200 µm (99.9% Metals basis - Alfa Aesar, Ward Hill, MA, U.S.A.) were cut (5.5 cm in length) and the insulated body of the wires was coated using at least two layers of heat-shrink polytetrafluoroethylene insulation tubing (PTFE, HS Sub-Lite-Wall, 0.02 ± 0.001 in, blackopaque). To facilitate connection with the potentiostat, a gold pin was soldered to one end of the electrode and this contact further coated with insulating connector paint (MG Chemicals, Burlington, ON, Canada). To allow the functionalization, the uninsulated end of the electrodes was cut to a final length of 3.5 mm prior to electrochemical cleaning with the following protocol: (1) 1000-1500 cycles between −1 and −1.6 V in a solution of 0.5 M NaOH at 1 V s^{-1} to remove any residual thiol/organic contaminants on the electrode surface and (2) pulsed between 0 and 2 V for at least 16,000 cycles with a pulse length of 20 ms (no waiting between pulses) in 0.5 M H_2SO_4 to increase the electrode roughness, as previously reported.[6]

Electrode functionalization. For the EAB sensors (Figure 3, 4, 5, S5, and S6), we first reduced the thiol-modified 1C variant (100 µM) by treating it (2 µL) for 1 h in a solution of 10 mM tris(2-carboxyethyl)-phosphine hydrochloride (TCEP, 4 µL) at room temperature in the dark. The reduced aptamer was then dissolved in "assembling buffer" (10 mM Na_2HPO_4 with 1 M NaCl and 1 mM $MgCl_2$ at pH 7.3) at a final concentration of 200 nM. The electrochemically cleaned wire gold electrodes were then immersed in 500 µL of this solution for 1 h in the dark. Following this the electrode surface was rinsed with distilled water and incubated overnight in assembling buffer containing 5 mM 6-mercaptohexanol at room temperature in the dark, followed by a rinse with distilled water before use. Of note, during the modification steps the solutions have to cover all the area of uninsulated gold electrode.

Electrochemical experiments. Electrochemical measurements were performed at room temperature using a CHI1030C Multiplexer (CH Instruments, Austin, TX, USA) and a standard three-electrode cell containing a platinum counter electrode and an Ag/AgCl (3 M KCl) reference electrode. Square Wave Voltammetry (SWV) was performed using a potential window of -0.12 to -0.46 V, a potential step of 0.001 V and 0.025 V amplitude.

Binding curves. Experimental binding curves were performed in 9 mL of working buffer (137 mM NaCl, 2.7 mM KCl, 10 mM Na_2HPO_4, 1.8 mM KH_2PO_4, $MgCl_2$ 2 mM, at pH 7.4) (Figure 3, 4, and S6) or in a solution made of 4.5 mL of artificial saliva and 4.5 mL of dilution buffer (137 mM NaCl, 2.7 mM KCl, 10 mM Na_2HPO_4, 1.8 mM KH_2PO_4, $MgCl_2$ 4 mM, at pH 7.4; for a total volume of 9 mL) (Figure 5B), or in 9 mL of fetal bovine serum (Figure 5A). For each test we used at least three EAB wire sensors modified with the selected C1 aptamer sequence, and we collected the SWV signals using 5 Hz and 300 Hz frequencies. Initially, in absence of the target or interferents, we performed a preliminary treatment by interrogating the sensors with 40-80 scans until stable peak currents were obtained. Once the sensor's signal was stable increasing concentrations of the selected proteins was added to the testing solution at 15 min intervals and the sensors were interrogated.

The peak current for each sensor at each concentration of target was extracted using a script written in Python[7] which allow to perform the analysis of voltammetric data by subtracting the baseline current from the peak maxima ($I_{C1Variant}$). The resultant data were fitted using a Langmuir equation (single-site binding)[8] in Kaleidagraph (Synergy Software):

$$I_{C1Variant}^{[\mathrm{Target}]} = I_{C1Variant}^{0} + \left(\frac{[\mathrm{Target}]\left(I_{C1Variant}^{\mathrm{Target-MAX}} - I_{C1Variant}^{0}\right)}{[\mathrm{Target}] + K_D}\right) \quad \text{Eq. 3}$$

Here [Target] is the target concentration, $I_{C1Variant}^{[\mathrm{Target}]}$ is the raw signal current in the presence of protein target, $I_{C1Variant}^{0}$ is the background raw current seen in the absence of the target, $I_{C1Variant}^{\mathrm{Target-MAX}}$ is the raw current signal change seen at saturating target, and K_D *i*s the dissociation constant of the surface-bound aptamer.

Using the , $I_{C1Variant}^{0}$estimated from the fit we converted the raw signaling current of each sensor into relative signal change ($I_{\%}$) using the following equation:[9,10]

$$I_{C1Variant}^{\%\mathrm{Signal}} = \left(\frac{I_{C1Variant}^{[\mathrm{Target}]} - I_{C1Variant}^{0}}{I_{C1Variant}^{0}}\right) \cdot 100 \quad \text{Eq. 4}$$

Frequency Map. We determined the frequency dependence of the sensor's signal gain (relative signal change upon challenge with saturating target; Figure S5) by interrogating three sensors in the absence and in the presence of a saturating concentration of RBD protein (100 nM). The collected peak currents were converted in signal gain using equation (4), where $\boldsymbol{I^0_{C1Variant}}$ correspond to the current in the absence of target.[11,12]

Sensor equilibration time. We determined the sensor's equilibration time (Figure 4B) using the above experimental approach and interrogating the sensor every 15 s at 5Hz and 300 Hz in working buffer (137 mM NaCl, 2.7 mM KCl, 10 mM Na_2HPO_4, 1.8 mM KH_2PO_4, $MgCl_2$ 2 mM at pH 7.3). After we achieved a stable current baseline (5 min) we added to the solution 10 nM of RBD and 10 nM of S1 protein, and then we monitored the voltammetric signal for over 15 min. Using the previously cited Python script[7] we analyzed the data and using Eq.4 we converted the raw current in relative % signal change.

Supporting Figures

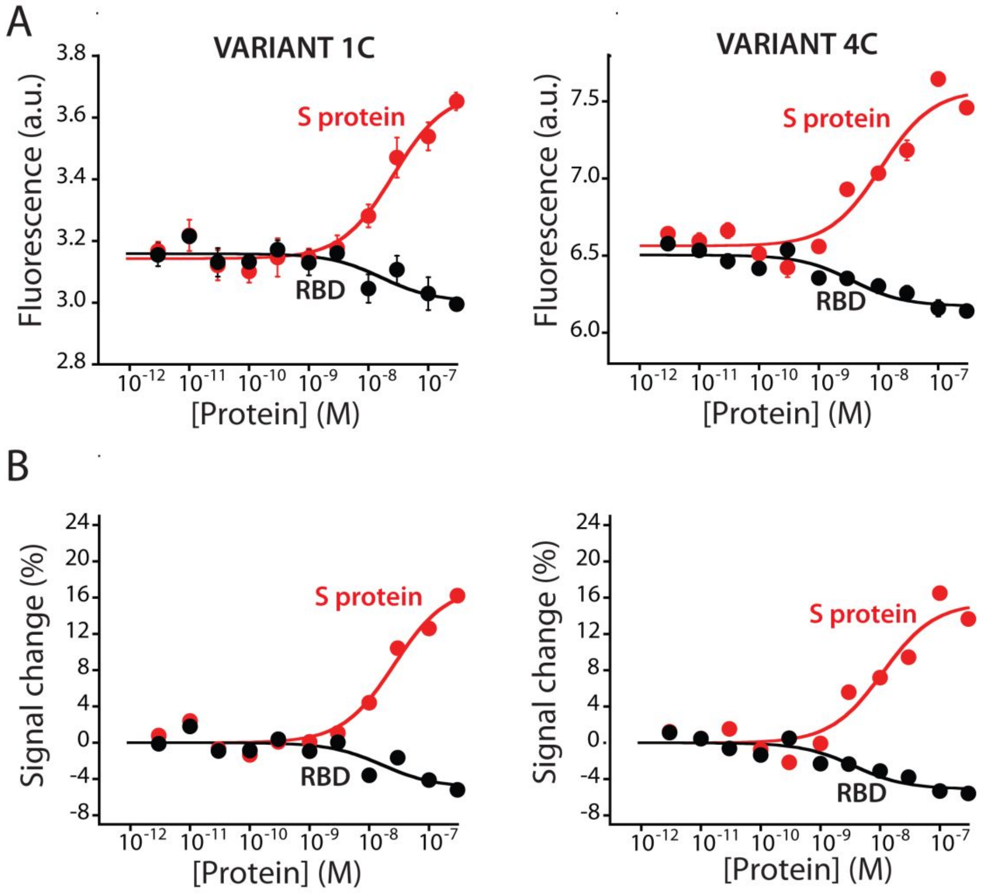

Figure S1. To characterize the binding activity and the structure-switching properties of the S protein-binding aptamers we labeled them using a fluorophore (6-FAM) and quencher (BHQ-1) at both ends (5' and 3', respectively). Using binding curves we characterized their affinities for the RBD (black) and S protein (red) and we converted (A) the raw fluorescence signal to (B) signal change (%) (see Material and Methods section). We found 1C variant responds to the S protein and RBD with a comparable signal gain and dissociation constant (K_D) to 4C variant. Binding curves were obtained in PBS (NaCl 137 mM, KCl 2.7 mM, Na_2HPO_4 10 mM, KH_2PO_4 1.8 mM), $MgCl_2$ 2 mM at pH 7.4, at 25°C using a concentration of aptamer of 5 nM.

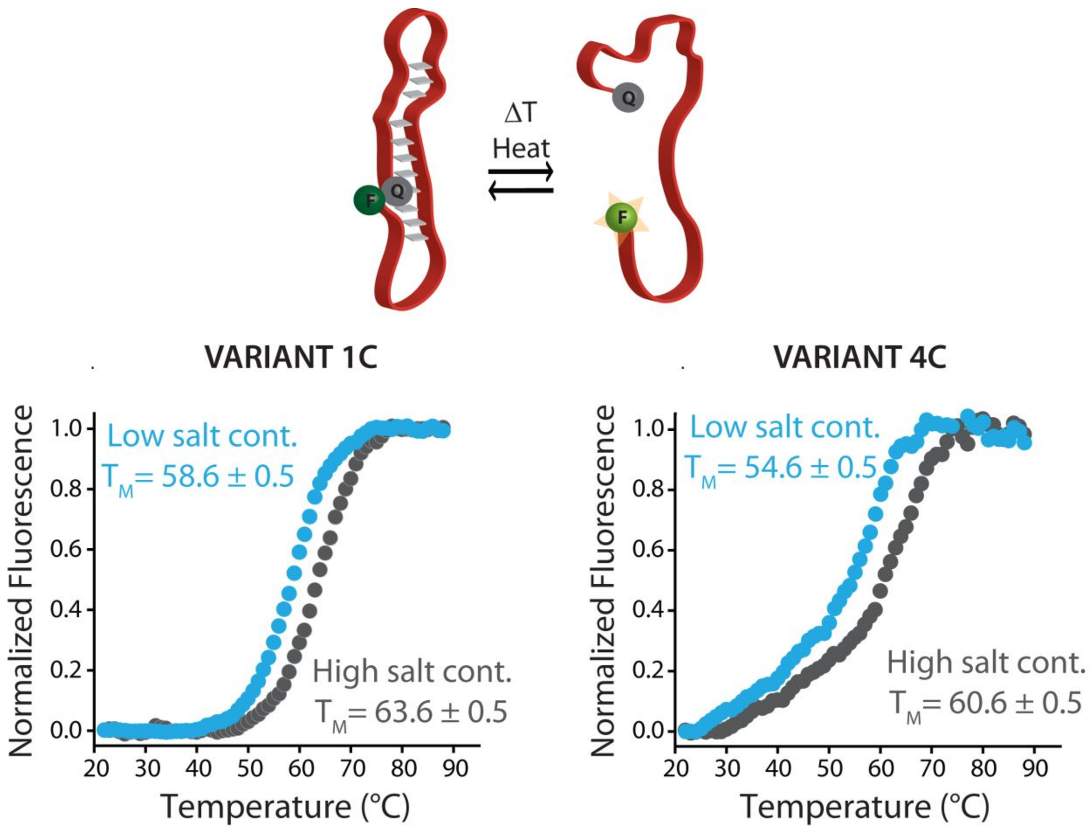

Figure S2. Normalized thermal melting curves for the 1C and 4C variant performed in the working buffer (gray curves) and in the newly buffer at lower salts concentrations (blue curves) used to destabilize the native conformation of the aptamers (See Material and Methods for details). Both variants display the classic melting transition which can be analyzed for the estimation of the melting temperatures (Tm). As expected, the melting curves performed in a buffer with a low amount of salts display lower values than the curves performed in the working buffer. Melting curves in gray were obtained in PBS (NaCl 137 mM, KCl 2.7 mM, Na_2HPO_4 10 mM, KH_2PO_4 1.8 mM), $MgCl_2$ 2 mM at pH 7.4, while melting curves in blue were obtained in 0.1x PBS (NaCl 13.7 mM, KCl 0.27 mM, Na_2HPO_4 1 mM, KH_2PO_4 0.18 mM), $MgCl_2$ 2 mM, at pH 7.4 and using a concentration of aptamer of 5 nM.

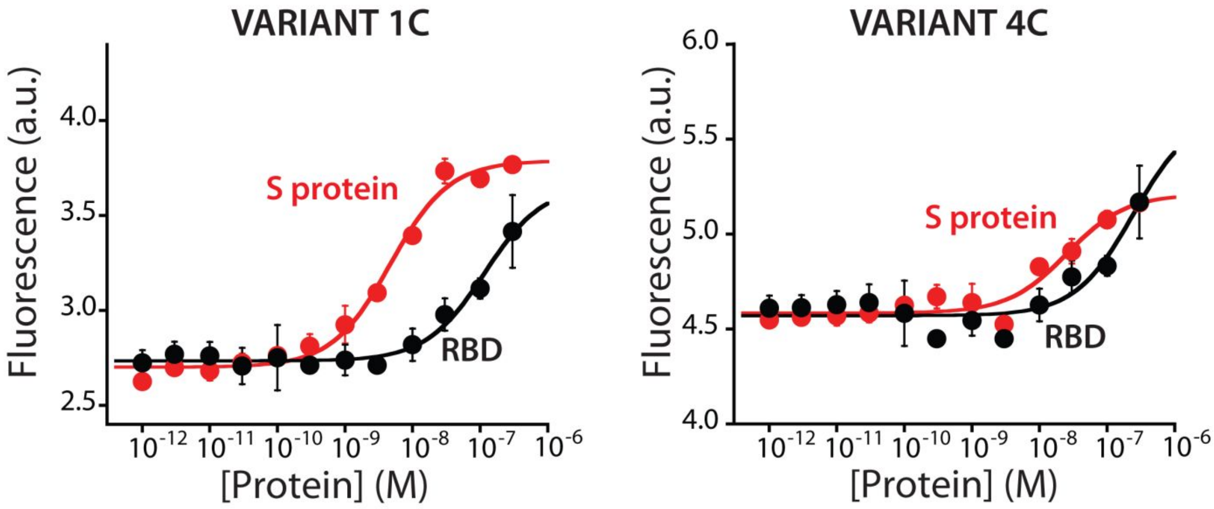

Figure S3. We characterized the binding activity of the 1C (left) and 4C (right) optically labeled variant in the new buffer at lower ionic salts content. When the aptamers are challenged against RBD (black) and S protein (red) they can promptly respond to the presence of the target proteins displaying a higher signal change and lower affinities (compared to the working buffer). The collected data demonstrate the ability of the aptamers to bind their targets through a conformational change and their potential to support EAB sensing platform. Binding curves were obtained in PBS (NaCl 13.7 mM, KCl 0.27 mM, Na_2HPO_4 1 mM, KH_2PO_4 0.18 mM), $MgCl_2$ 2 mM at pH 7.4, at 25°C using a concentration of aptamer of 5 nM.

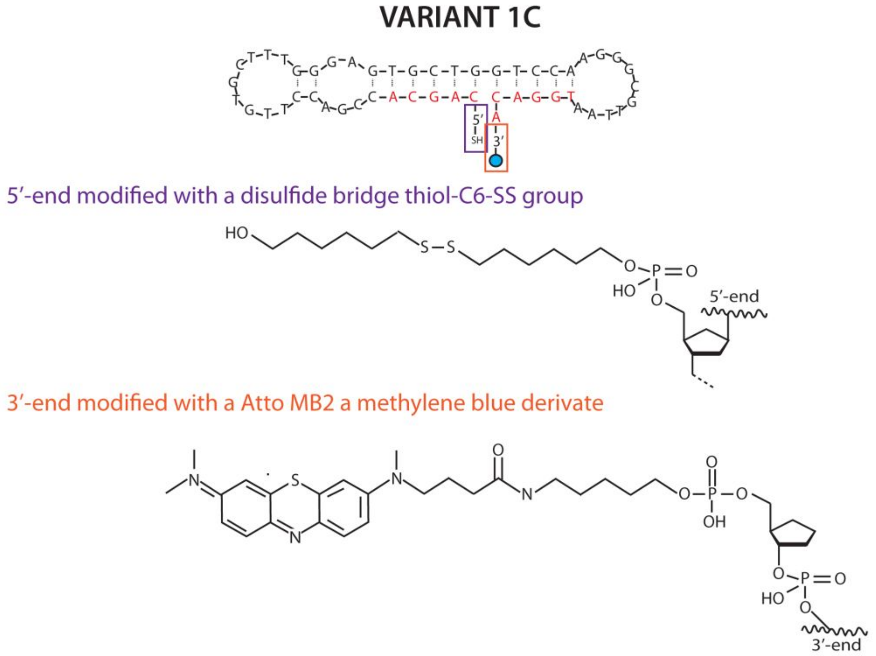

Figure S4. Sequence of the selected aptamer and structure of the thiolated (purple) and Atto MB2 (orange) modifications employed in the fabrication of EAB sensor.

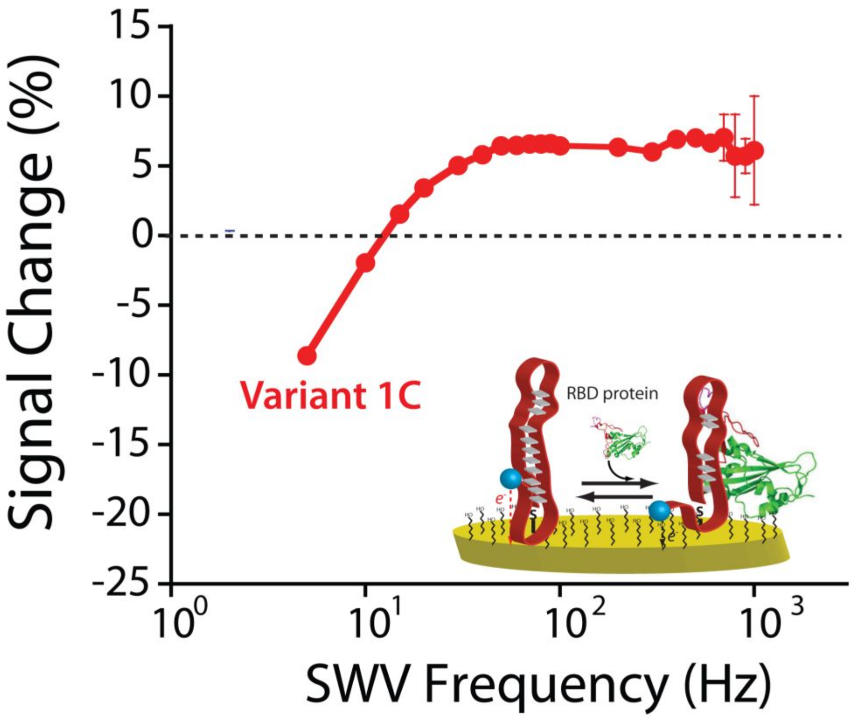

Figure S5. To demonstrate the binding-induced conformational change of the aptamer on the electrode surface, we characterized the relationship between its signal change and the frequency of the interrogating SWV potential pulse. Here we have tested the sensor over a range of square-wave frequencies (from 5 Hz to 1000 Hz) in the absence and in presence of saturating concentration of RBD protein (100 nM). Shown is the relative gain (relative signal change between zero and saturating target).

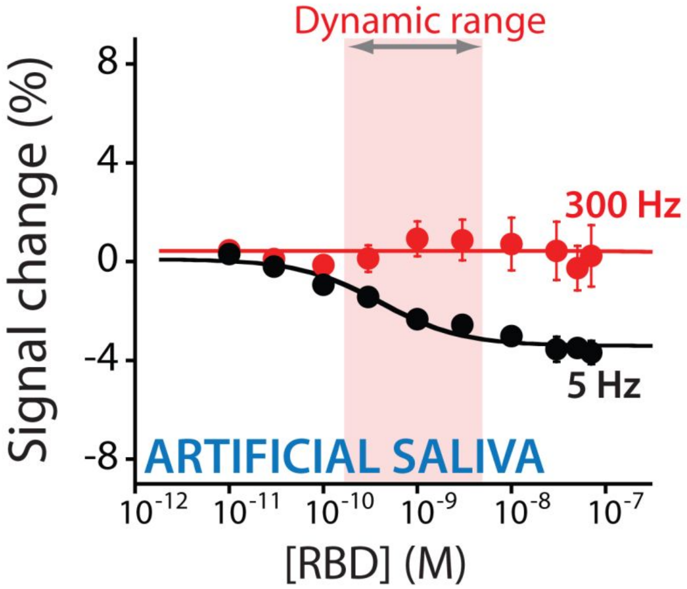

Figure S6. We characterized the response of our EAB sensor directly in 100% artificial saliva against RBD protein. Despite the sensor promptly respond to the presence of the target at low frequency, its overall signal gain is lower because there is no signal change at high frequency.

References

(1) biomers.net | Electrochemical detection - biomers.net Oligonucleotides https://www.biomers.net/en/products/dna/electrochemical_detection.html#Atto MB2 (accessed Apr 20, 2021).

(2) Song, Y.; Song, J.; Wei, X.; Huang, M.; Sun, M.; Zhu, L.; Lin, B.; Shen, H.; Zhu, Z.; Yang, C. Discovery of Aptamers Targeting the Receptor-Binding Domain of the SARS-CoV-2 Spike Glycoprotein. *Anal. Chem.* **2020**, *92* (14), 9895–9900. https://doi.org/10.1021/acs.analchem.0c01394.

(3) Ricci, F.; Vallée-Bélisle, A.; Simon, A. J.; Porchetta, A.; Plaxco, K. W. Using Nature's "Tricks" to Rationally Tune the Binding Properties of Biomolecular Receptors. *Acc. Chem. Res.* **2016**, *49* (9), 1884–1892. https://doi.org/10.1021/acs.accounts.6b00276.

(4) Mergny, J. L.; Lacroix, L. Analysis of Thermal Melting Curves. *Oligonucleotides* **2003**, *13* (6), 515–537. https://doi.org/10.1089/154545703322860825.

(5) Idili, A.; Ricci, F.; Vallée-Bélisle, A. Determining the Folding and Binding Free Energy of DNA-Based Nanodevices and Nanoswitches Using Urea Titration Curves. *Nucleic Acids Res.* **2017**, *45* (13), 7571–7580. https://doi.org/10.1093/nar/gkx498.

(6) Arroyo-Currás, N.; Scida, K.; Ploense, K. L.; Kippin, T. E.; Plaxco, K. W. High Surface Area Electrodes Generated via Electrochemical Roughening Improve the Signaling of Electrochemical Aptamer-Based Biosensors. *Anal. Chem.* **2017**, *89* (22), 12185–12191. https://doi.org/10.1021/acs.analchem.7b02830.

(7) Curtis, S. D. S. D.; Ploense, K. L.; Kurnik, M.; Ortega, G.; Parolo, C.; Kippin, T. E. T. E.; Plaxco, K. W. K. W.; Arroyo-Currás, N. Open Source Software for the Real-Time Control, Processing, and Visualization of High-Volume Electrochemical Data. *Anal. Chem.* **2019**, *91* (19), 12321–12328. https://doi.org/10.1021/acs.analchem.9b02553.

(8) Esteban Fernández De Ávila, B.; Watkins, H. M.; Pingarrón, J. M.; Plaxco, K. W.; Palleschi, G.; Ricci, F. Determinants of the Detection Limit and Specificity of Surface-Based Biosensors. *Anal. Chem.* **2013**, *85* (14), 6593–6597. https://doi.org/10.1021/ac4012123.

(9) Idili, A.; Parolo, C.; Ortega, G.; Plaxco, K. W. Calibration-Free Measurement of Phenylalanine Levels in the Blood Using an Electrochemical Aptamer-Based Sensor Suitable for Point-of-Care Applications. *ACS sensors* **2019**, *4* (12), 3227–3233. https://doi.org/10.1021/acssensors.9b01703.

(10) Idili, A.; Gerson, J.; Kippin, T.; Plaxco, K. W. Second-Resolved, in-Situ Measurements of Plasma Phenylalanine Disposition Kinetics in Living Rats. *Anal. Chem.* **2021**, *93* (8), 4023–4032. https://doi.org/http://dx.doi.org/10.1021/acs.analchem.0c05024.

(11) White, R. J.; Plaxco, K. W. Exploiting Binding-Induced Changes in Probe Flexibility for the Optimization of Electrochemical Biosensors. *Anal. Chem.* **2010**, *82* (1), 73–76. https://doi.org/10.1021/ac902595f.

(12) Idili, A.; Amodio, A.; Vidonis, M.; Feinberg-Somerson, J.; Castronovo, M.; Ricci, F. Folding-upon-Binding and Signal-on Electrochemical DNA Sensor with High Affinity and Specificity. *Anal. Chem.* **2014**, *86* (18), 9013–9019. https://doi.org/10.1021/ac501418g.
